# Genetic characterization of equid herpesvirus type 1 from cases of abortion in Poland

**DOI:** 10.1007/s00705-017-3376-3

**Published:** 2017-04-27

**Authors:** Karol Stasiak, Magdalena Dunowska, Simon F. Hills, Jerzy Rola

**Affiliations:** 1 Department of Virology, National Veterinary Research Institute, Al. Partyzantow 57, 24-100 Pulawy, Poland; 2 Institute of Veterinary, Animal and Biomedical Sciences, Massey University, Palmerston North, New Zealand; 3 Institute of Agriculture and Environment, Massey University, Palmerston North, New Zealand

## Abstract

Equid herpesvirus type 1 (EHV-1) is a common viral infection associated with varied clinical outcomes including respiratory disease, abortion and neurological disease. We have characterized EHV-1 sequences (n = 38) obtained from cases of equine abortion in Poland between 1999 and 2016, based on sequencing of PCR products from open reading frames (ORF) 30 and 68 of the EHV-1 genome. The majority (81.6%) of sequences were not classified into any of the previously described groups based on the ORF68 sequence. The remaining sequences belonged to ORF68 group III (7.9%) or IV (10.5%). A haplotype network analysis did not show any obvious structure within networks of local Polish sequences, nor within a global network of 215 EHV-1 sequences when these networks were coloured based on the geographical origin of viruses or date of detection. Our data suggest that ORF68 does not provide a reliable molecular marker for epidemiological studies of EHV-1, at least in a global sense. Its usefulness to aid local investigations of individual outbreaks remains to be established. All but two Polish EHV-1 sequences belonged to the ORF30 N_752_ genotype. The two ORF30 D_752_ viruses were obtained from abortion cases in 2009 and 2010. Hence, abortion cases that occurred in Poland between 1999 and 2016 were caused predominantly by EHV-1 with the ORF30 N_752_ genotype, with no indication of an increase in the prevalence of the ORF30 D_752_ variant.

**Electronic supplementary material:**

The online version of this article (doi:10.1007/s00705-017-3376-3) contains supplementary material, which is available to authorized users.

## Introduction

Equid herpesvirus 1 (EHV-1) is a common pathogen of horses worldwide [1]. Of the five currently recognised equine herpesviruses [2], EHV-1 is considered to be the most important due to its potential for high emotional and economic impact [3]. Many EHV-1 infections are subclinical, but the virus can also cause respiratory disease of varying severity, abortion, neonatal death or neurological disease [4]. The frequency of reports of neurological disease associated with EHV-1 infection (equine herpesvirus myeloencephalopathy, EHM) seems to have increased in some parts of the world over the past 10-15 years, causing concerns among horse owners and veterinarians [5].

While the existence of EHV-1 viruses with different pathogenic potential is well recognised [6–8], factors that influence the clinical outcome of EHV-1 infection are poorly understood. A feature of EHV-1 that seems to be directly linked to its virulence is the ability of the virus to establish cell-associated viraemia [3, 5, 9]. Highly virulent variants of EHV-1 seem to be able to establish cell-associated viraemia of higher magnitude than variants of lower virulence [10, 11].

One of the proposed markers associated with increased virulence is dimorphism in the nucleotide sequence of the DNA polymerase gene encoded by open reading frame (ORF) 30. A single amino acid substitution from asparagine (N) to aspartic acid (D) at position 752 has been associated with increased neurovirulence [10, 12]. However, this relationship is not observed for all EHV-1 field viruses, as is exemplified by the fact that in a recent Australian study, four out of five archival EHV-1 isolates from EHM cases belonged to the ORF30 N_752_ genotype [13]. Hence, it is likely that the viral markers of virulence are more complex than this single amino acid substitution [5, 14].

Considering the potential economic and emotional impact of EHV-1 infections, it would be beneficial to understand the local epidemiology and molecular evolution of these viruses. This would increase our knowledge about the ways they spread within a given facility, within a given region, or across larger geographical areas. It would also facilitate tracking the sources of EHV-1 in an outbreak situation. Such information could then be utilised for the development of effective control and prevention strategies. However, the genome of EHV-1 appears to be relatively stable, with very little variability, even between viruses with markedly different disease potential. For example, only approximately 0.1% variation at the nucleotide level over the entire genomic sequence has been detected between two well-characterized EHV-1 strains of different virulence: Ab4 and V592 [12]. A short region spanning approximately 600 bp of ORF68 has been proposed by the same authors to be a putative genetic marker that may be useful for epidemiological studies. Based on single-nucleotide polymorphisms (SNPs) observed in this region, 106 EHV-1 sequences of field viruses were clustered into six groups [12]. The grouping appeared to reflect the geographical origins of the viruses. Subsequently, the same region within ORF68 was used for molecular comparison of EHV-1 from Hungary [15] and Australia [13], with conflicting conclusions regarding the usefulness of the system for molecular tracking of EHV-1.

The aim of the current study was to characterize Polish EHV-1 sequences based on ORF68 SNPs in order to add to the existing data from other countries. Specifically, we were hoping to determine whether or not this classification system was applicable for molecular tracking of Polish EHV-1 viruses, both within the local (within the country) and global (between countries) sense.

## Materials and methods

### Source of samples

**Fig. 1:**
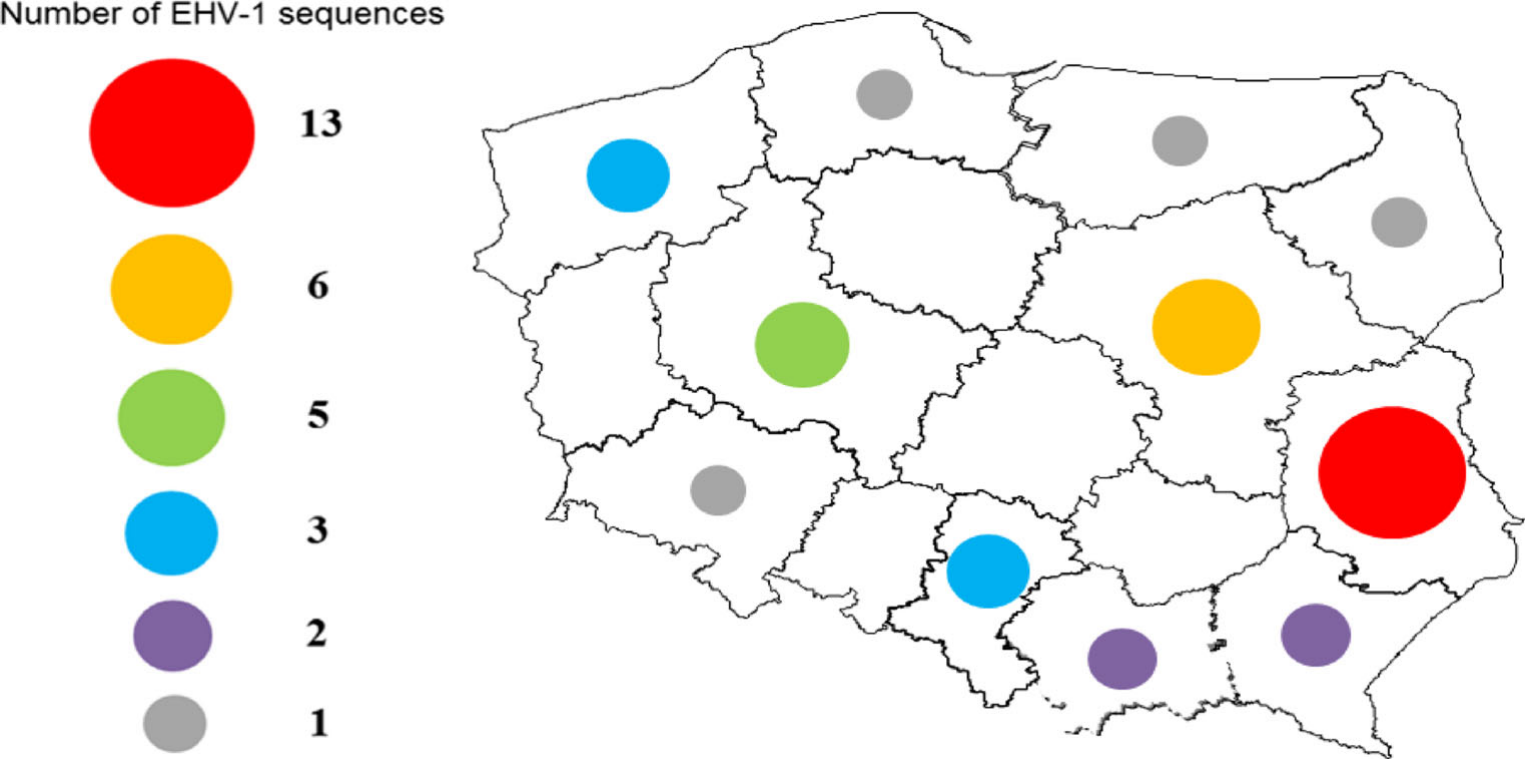
Sources of Polish EHV-1 sequences included in the study, stratified by province of origin

The viruses (n = 29) used in this study were isolated in RK13 cells from tissue homogenates from cases of equine abortion that had been submitted by field veterinarians to the Department of Virology of the National Veterinary Research Institute in Pulawy (Poland) for EHV-1 testing. In addition, EHV-1 PCR-positive tissue homogenates (n = 9) that were negative for virus isolation during the initial investigation were also included. Altogether, these 38 samples comprised all EHV-1-positive samples identified from a total of 109 submissions received between 1999 and 2016, and originated from small private stables and national horse studs located throughout Poland (Fig. 1).

The tissue types submitted for investigation varied but typically included fetal spleen, liver, and lung. Some submissions also included heart, kidney, thymus and placenta. All tissues were sent to the laboratory on ice packs and were typically received within 1-3 days of collection. On arrival in the laboratory, the tissues were processed according to standard laboratory protocols at the time of submission. Typically, 10% (w/v) homogenates in Eagle’s minimum essential medium (MEM, Sigma-Aldrich) supplemented with 1% antibiotic-antimycotic solution (Sigma-Aldrich) were prepared from each tissue. Homogenates from all tissue types submitted from the same case were then pooled, clarified by low-speed centrifugation, filtered through a 0.45-µm filter, and used for virus isolation and PCR as described previously [16, 17]. None of the isolates were passaged in cell culture more than three times.

Samples submitted between 1999 and 2012 were tested for both EHV-1 and EHV-4 using conventional PCR [18], while samples submitted in 2013 onwards were tested using quantitative PCR (qPCR) [19]. Both assays targeted the glycoprotein B (gB) gene. Viral isolates and EHV-1-positive tissue homogenates were stored at -80 °C.

### Processing of samples

Total DNA was extracted from EHV-1 isolates and from EHV-1-positive tissue homogenates using a High Pure PCR Template Preparation Kit (Roche Diagnostics GmbH, Mannheim, Germany) according to the manufacturer’s instructions.

PCR amplification of a 764-bp fragment of EHV-1 ORF68 was based on a protocol described by Malik et al. [15]. Each PCR reaction consisted of 0.6 mM deoxynucleotide mix (Sigma-Aldrich), 0.5 µL JumpStart AccuTaq LA DNA Polymerase (Sigma-Aldrich), 2.5 mM MgCl_2,_ 2.5 µL of DMSO, 0.4 µM each primer (ORF68f, TTGGCATCTGAACCGCTTGG; ORF68r, AGAGTAGGCGTTCCATCCAC) and 2 µL of template DNA in 1x buffer (Sigma-Aldrich) in a total volume of 25 µL. Amplifications were performed in a Biometra Thermocycler (Biometra, Germany) using the following cycling conditions: 3 minutes of initial denaturation at 95 °C, followed by 40 cycles of denaturation (1 minute at 95 °C), annealing (1 minute at 60.8 °C) and elongation (2 minutes at 72 °C).

A 380-bp product from the viral DNA polymerase gene was amplified and digested with SalI as described previously [16], using template DNA from samples (n = 18) that had not been previously assigned to N/D_752_ variants. The viruses were assigned to N/D_752_ variants based on restriction fragment length polymorphism (RFLP) of the digested product.

PCR products of the expected sizes from both ORF30 and ORF68 PCR were sequenced using BigDye^®^ Terminator version 3.1 (Applied Biosystems) on a 3730xl DNA Analyzer (Applied Biosystems) at Genomed (Warsaw, Poland). The obtained sequences were assembled using BioEdit software (version 7.2.5). Alignment and comparison of the nucleotide sequences were carried out using ClustalW in MEGA version 5.0.5. [20].

### Network analysis

A total of 37 EHV-1 ORF68 PCR products that generated good-quality sequence data were used for phylogenetic analysis (the sequence from PL_2015_I was of insufficient quality). Sequences were aligned in Geneious v 9.1.3, and ambiguous base calls where resolved by reference to chromatographs. For each sequence, a consensus sequence was generated from the forward and reverse sequencing products. Additional EHV-1 sequences (n = 178) originating from various countries (including one Polish sequence) were obtained from the National Centre for Biotechnology Information (NCBI) database. Selected sequences had an ORF68 annotation, were at least 500 bp in length, and were not the only representative from a given country. An alignment of all 215 EHV-1 ORF68 sequences (464 bp) was generated in Geneious v 9.1.3, and exported in nexus file format for downstream analysis.

Population structure analysis was performed in PopART version 1.6 (available from http://popart.otago.ac.nz) using default parameters to produce median-joining haplotype networks. Sequences were grouped based on areas of origin in order to test for geographic clustering of genotypes. Analysis of molecular variance was carried out in PopART using the ‘Simple AMOVA’ command.

### GenBank accession numbers

The nucleotide sequences of the Polish EHV-1 isolate described in this study were deposited in GenBank under the accession numbers KY201117-KY201134 (ORF30) and KY201135-KY201172 (ORF68).

## Results

### ORF30 genotypes

All of the viruses tested as part of the current study belonged to the ORF30 N_752_ variant genotype based on PCR-RFLP testing, which was also confirmed by sequencing.

### ORF68 genotypes

**Fig. 2:**
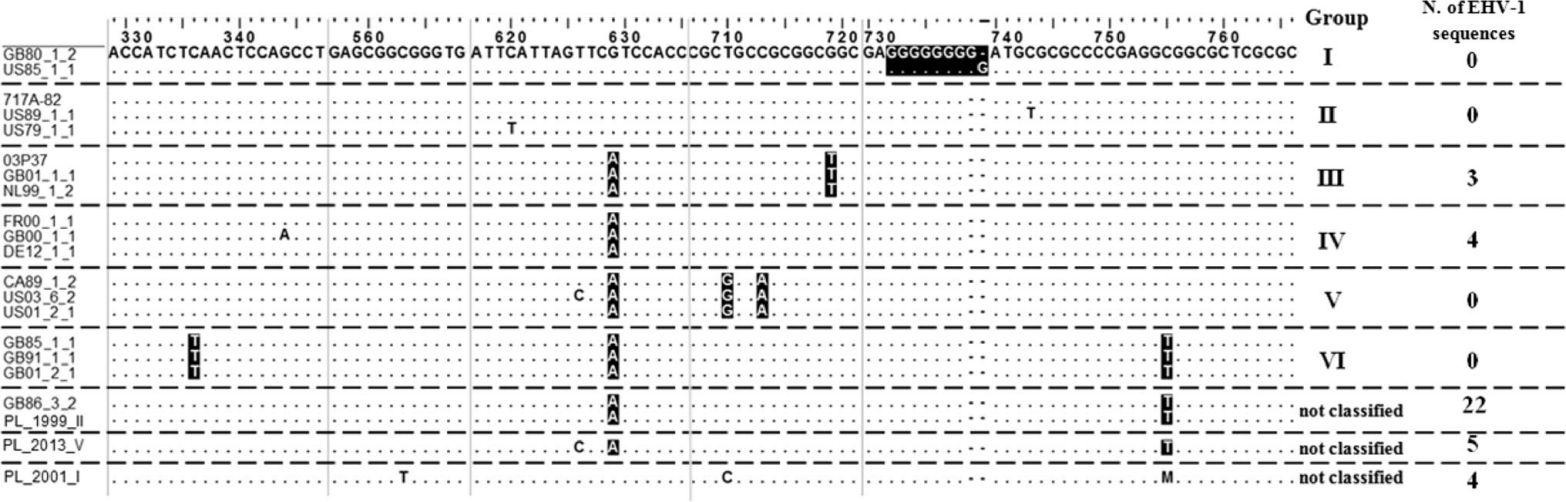
Location of SNPs within the ORF68 gene of Polish EHV-1 sequences. Nucleotides are numbered according to accession number DQ172353.1. Nucleotide positions typical for each of the ORF68 groups [12] are highlighted

Out of 38 Polish EHV-1 sequences analyzed in the current study, three (7.9%) belonged to group III, four (10.5%) belonged to group IV, and the remaining 31 (81.6%) were not classified within any of the groups originally described by Nugent and colleagues [12] (Fig. 2). Interestingly, over half of Polish EHV-1 sequences (57.9%) contained A_629_ and T_755_ SNPs. The same substitutions were also present in the EHV-1 sequence from UK-GB86_3_2, which was reported as an unassigned sequence in the original study by Nugent et al. [12]. All Polish EHV-1 sequences from the current study contained seven G residues in a homopolymeric tract (nt 732 to 739).

### Network analysis

**Fig. 3:**
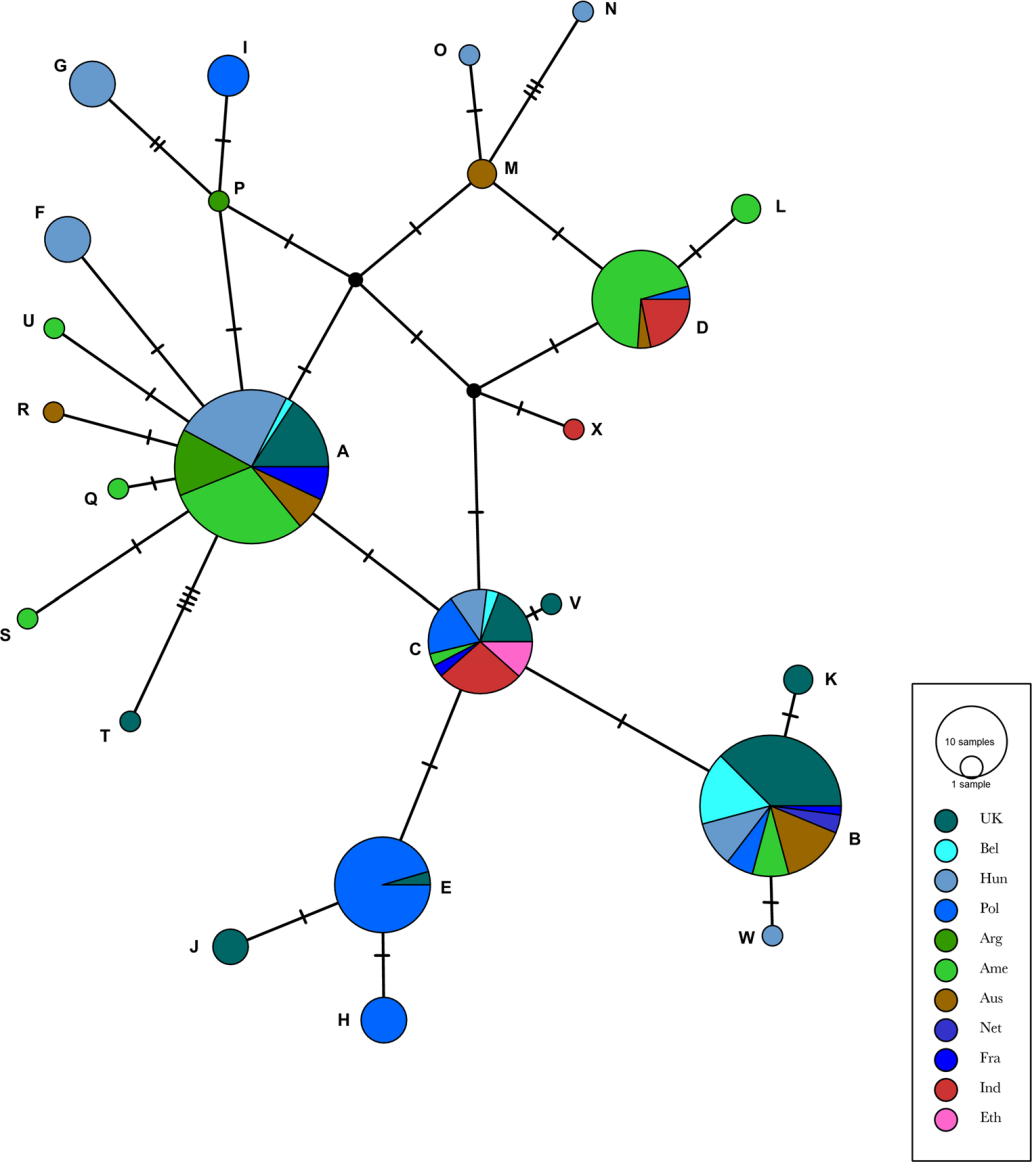
International network of EHV-1 ORF68 sequences including sequences from GenBank (n = 178) and Polish sequences described in the current study (n = 37, labelled PL_year of isolation_ID number). All sequences were concatenated and trimmed to 464 nt. Nodes are labelled with capital letters (A through F), scaled based on the number of representative sequences, and coloured based on the geographic origin of the sample (country). The details for sequences included in each node are listed in Online Resource 1. No obvious clustering was evident in the network shown in the figure. There was also no clustering when the network was coloured by the date of EHV-1 detection

**Fig. 4:**
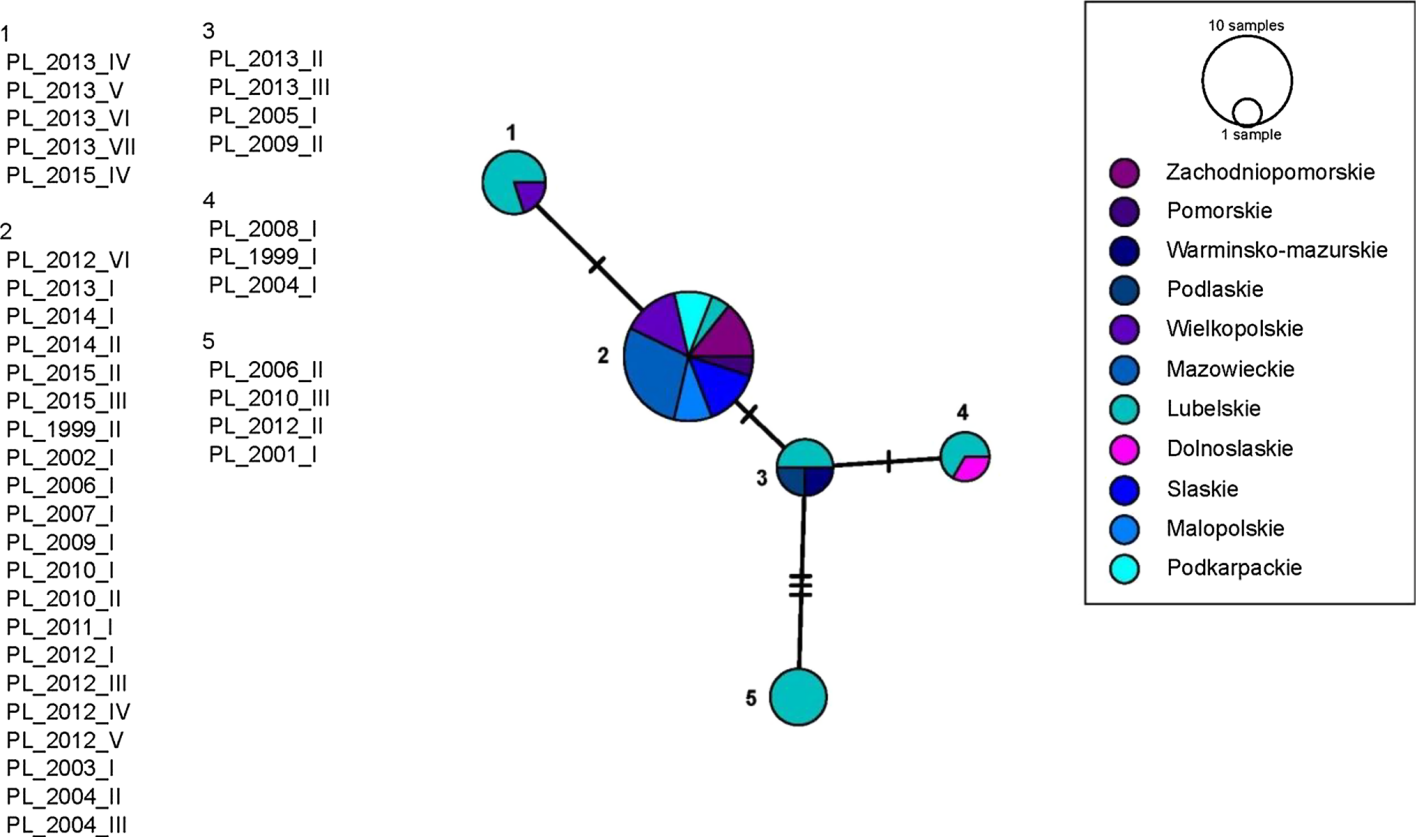
Haplotype network of EHV-1 ORF68 sequences from Poland (n = 37, labelled PL_year of isolation_ID number). All sequences were concatenated and trimmed to 688 nt. Nodes are labelled with numbers (1 through 4), scaled based on the number of representative sequences, and coloured based on the geographic origin of the sample (province). No obvious clustering was evident in the network

There was no obvious structure in any of the networks generated. Genetic variation in ORF68 was not strongly correlated with geographic distribution (ϕ_st_ = 0.20646, *p* < 0.001) based on country of origin (Fig. 3 and Online Resource 1). While some smaller nodes consisted predominantly of sequences from one country (for example, nodes E and H contained Polish sequences), the two largest nodes (A and B) included viruses from a variety of geographical regions. Similarly, there was no apparent structure in the network of local Polish EHV-1 sequences colored by the geographical region in Poland (province) from which they originated (Fig. 4).

There was also no structure in the global network when sequences were colored by date of origin. Often, very similar sequences from a single node span several decades, as seen, for example, in node A. Similarly, the dates of isolation of Polish EHV-1 sequences from node E (Fig. 3) span 16 years (1999 to 2015). At the same time, some viruses from the same year/country clustered in several different nodes (e.g., Polish viruses from 2013 clustered within three different nodes).

## Discussion

The aim of the current study was to characterize Polish EHV-1 viruses based on ORF30 and ORF68 sequence data. It has been shown previously that 2/20 Polish EHV-1 isolates from samples submitted between 1999 and 2012 belonged to the ORF30 D_752_ variant genotype [16]. The two D_752_ EHV-1 viruses were detected from abortion cases in 2009 and 2010. This, combined with the fact that all viruses in the current study belonged to the ORF30 N_752_ genotype, suggests that the ORF30 D_752_ variant, while present in Poland, is not common. Our results are in contrast to those reported from several other countries, where the frequency of detection of ORF30 D_752_ EHV-1 seems to have increased over the past decade or so [21, 22]. This apparent increase in the frequency of detection of the ORF30 D_752_ variant seems to parallel the perceived increase in reports of EHM cases in the USA and several European countries [5]. However, a similar increase in the number of reported EHM cases has not been observed in Poland, nor in several other countries such as Australia [13] or New Zealand [23]. The lack of detection of the ORF30 D_752_ variant in samples collected between 2012 and 2016 in the current study corresponds well to the lack of reports of EHM in Polish horses. Alternatively, it may also reflect the relatively small sample size or the source of EHV-1 (abortion versus EHM cases). EHM may truly be rare among Polish horses, or it may be underreported due to inherent difficulties associated with this diagnosis, particularly in sporadic cases [24]. The suitability of the amplified ORF68 region as a molecular marker associated with the geographical origin of the virus was first proposed by Nugent et al. [12], who established six ORF68 groups (I to VI) based on SNPs present within this short (559 bp) region. A similarly large amount of variability in the location of SNPs within the analyzed ORF68 region was also reported in a recent Hungarian study, where four additional groups were established to accommodate all ORF68 SNP patterns from 35 Hungarian EHV-1 isolates [15].

Consistent with the results of these previous studies, 37 Polish EHV-1 formed five nodes (Fig. 3) based on ORF68 analysis. The majority (21/37, 56%) of the Polish EHV-1 sequences analyzed clustered together in a global network (node E in Fig. 3), despite the fact that the dates of isolation of these viruses span 16 years (1999 to 2015). However, the results of the network analysis did not support the use of ORF68 as an epidemiological tool for monitoring the origin and geographical spread of EHV-1, as there was no obvious structure in any of the networks generated. Similar conclusions have recently been reached by Australian investigators, based on the analysis of 66 archival Australian EHV-1 sequences [13]. The existence of several different ORF68 genotypes within the same geographical region of Hungary was also reported by Malik et al. [15]. Interestingly, sequences with SNPs characteristic of group II viruses were most frequently (40%) detected among the 35 Hungarian EHV-1 isolates tested in that study, with only six (17.1%) of the sequences classified as belonging to group III. This was in contrast to data from the original paper by Nugent et al. [12], who reported that group II sequences were most common among EHV-1 isolates of American origin, and group III sequences were most common among EHV-1 isolates of European origin.

Four viruses from the same outbreak of EHV-1-associated abortion that occurred in 2013 (PL_2013_IV to VII) all grouped together (group H in Fig. 3). To our knowledge, none of the other Polish viruses included in the current study were epidemiologically linked. This raises the possibility that, while ORF68 does not appear to be a useful marker in a global sense, it may facilitate tracking of EHV-1 within the outbreak situation and allow differentiation of the outbreak virus from other EHV-1 strains that may circulate locally. Availability of a larger data set including multiple sequences from recognized outbreaks of EHV-1-associated disease would be required to further evaluate this possibility.

In summary, based on the available data, the ORF68 does not appear, to provide a reliable molecular marker for epidemiological studies of EHV-1, at least in a global sense. Its usefulness for aiding local investigations of individual outbreaks remains to be established. Abortion cases that occurred in Poland between 1999 and 2016 were caused predominantly by viruses with the ORF30 N_752_ genotype, with no indication of the increase in the prevalence of the D_752_ variant.

## Electronic supplementary material

Below is the link to the electronic supplementary material.

Supplementary material 1 (DOC 306 kb)
