# Supplementary material for: Genetic characterization of equid herpesvirus type 1 from cases of abortion in Poland
Source: Arch Virol. 2017 Apr 27;162(8):2329–35. doi: 10.1007/s00705-017-3376-3 (PMC5506511; doi:10.1007/s00705-017-3376-3)
Supplement: Supplementary file 1 — Supplementary material 1 (DOC 306 kb) [file 705_2017_3376_MOESM1_ESM.doc]

Article title: Genetic characterization of equid herpesvirus type 1 from cases of abortion in Poland.

Journal name: Archives of Virology

Author names: Karol Stasiak ^1^ , Magdalena Dunowska ^2^, Simon F. Hills ^3^, Jerzy Rola* ^1^

^1^ Department of Virology, National Veterinary Research Institute, Pulawy, Poland

^2^ Institute of Veterinary, Animal and Biomedical Sciences, Massey University, New Zealand

^3^ Institute of Agriculture and Environment, Massey University, Palmerston North, New Zealand

*Corresponding author: jrola@piwet.pulawy.pl

Details for sequences included in the international network in Fig. 3

| **Haplotype** | **Genetic group^1^** | **Country of Origin^2^** | **NCBI Accession^3^** | **Sampling Year** | **Reference** |
| --- | --- | --- | --- | --- | --- |
| A | 1 | Arg | DQ172310 | 1985 | Nugent et al. |
| A | 2 | Arg | DQ172311 | 1990 | Nugent et al. |
| A | 2 | Arg | DQ172312 | 1990 | Nugent et al. |
| A | 2 | Arg | DQ172313 | 1991 | Nugent et al. |
| A | 2 | Arg | DQ172314 | 1991 | Nugent et al. |
| A | 2 | Arg | DQ172315 | 1991 | Nugent et al. |
| A | 2 | Arg | DQ172316 | 1996 | Nugent et al. |
| A | 2 | Arg | DQ172308 | 2000 | Nugent et al. |
| A | 2 | Aus | DQ172318 | 1982 | Nugent et al. |
| A | 2 | Aus | KT324733 | 1982 | Vaz et al. |
| A | 2 | Aus | KT324730 | 1994 | Vaz et al. |
| A | 2 | Aus | KT324725 | 2007 | Vaz et al. |
| A | 2 | Bel | GU271938 | 2009 | Gryspeerdt et al. |
| A | 1 | UK | NC_001491 | 1991 | Telford et al. |
| A | 2 | Fra | DQ172328 | 1997 | Nugent et al. |
| A | 2 | Fra | DQ172329 | 1999 | Nugent et al. |
| A | 2 | Fra | DQ172330 | 1999 | Nugent et al. |
| A | 2 | Fra | DQ172331 | 1999 | Nugent et al. |
| A | 2 | Hun | HQ654053 | 1977 | Malik et al. |
| A | 2 | Hun | HQ654058 | 1982 | Malik et al. |
| A | 2 | Hun | HQ654061 | 1988 | Malik et al. |
| A | 2 | Hun | HQ654062 | 1988 | Malik et al. |
| A | 2 | Hun | HQ654063 | 1990 | Malik et al. |
| A | 2 | Hun | HQ654065 | 1991 | Malik et al. |
| A | 2 | Hun | HQ654066 | 1992 | Malik et al. |
| A | 2 | Hun | HQ654080 | 1997 | Malik et al. |
| A | 2 | Hun | HQ654076 | 1998 | Malik et al. |
| A | 2 | Hun | HQ654077 | 1998 | Malik et al. |
| A | 2 | Hun | HQ654078 | 1998 | Malik et al. |
| A | 2 | Hun | HQ654070 | 2003 | Malik et al. |
| A | 2 | Hun | HQ654072 | 2004 | Malik et al. |
| A | 2 | Hun | HQ654082 | 2005 | Malik et al. |
| A | 2 | UK | DQ172352 | 1979 | Nugent et al. |
| A | 1 | UK | DQ172353 | 1980 | Nugent et al. |
| A | 1 | UK | DQ172356 | 1983 | Nugent et al. |
| A | 1 | UK | DQ172358 | 1983 | Nugent et al. |
| A | 2 | UK | DQ172360 | 1986 | Nugent et al. |
| A | 1 | UK | DQ172369 | 1993 | Nugent et al. |
| A | 2 | UK | DQ172370 | 1999 | Nugent et al. |
| A | 2 | UK | DQ172338 | 2003 | Nugent et al. |
| A | 2 | Ame | DQ172390 | 1970 | Nugent et al. |
| A | 2 | Ame | DQ172393 | 1976 | Nugent et al. |
| A | 2 | Ame | DQ172397 | 1983 | Nugent et al. |
| A | 2 | Ame | DQ172399 | 1984 | Nugent et al. |
| A | 1 | Ame | DQ172400 | 1985 | Nugent et al. |
| A | 2 | Ame | DQ172401 | 1985 | Nugent et al. |
| A | 2 | Ame | DQ172403 | 1985 | Nugent et al. |
| A | 2 | Ame | DQ172405 | 1986 | Nugent et al. |
| A | 2 | Ame | DQ172406 | 1986 | Nugent et al. |
| A | 2 | Ame | DQ172409 | 1990 | Nugent et al. |
| A | 2 | Ame | DQ172413 | 1999 | Nugent et al. |
| A | 2 | Ame | DQ172374 | 2000 | Nugent et al. |
| A | 2 | Ame | DQ172380 | 2003 | Nugent et al. |
| A | 2 | Ame | DQ172381 | 2003 | Nugent et al. |
| A | 2 | Ame | DQ172382 | 2003 | Nugent et al. |
| A | 2 | Ame | DQ172383 | 2003 | Nugent et al. |
| A | 2 | Ame | DQ172386 | 2003 | Nugent et al. |
| B | 3 | Aus | KT324734 | 1977 | Vaz et al. |
| B | 3 | Aus | KT324734 | 1977 | Vaz et al. |
| B | 3 | Aus | KT324732 | 1990 | Vaz et al. |
| B | 3 | Aus | DQ172317 | 2002 | Nugent et al. |
| B | 3 | Aus | KT324728 | 2002 | Vaz et al. |
| B | 3 | Aus | KT324729 | 2002 | Vaz et al. |
| B | 3 | Aus | KT324726 | 2007 | Vaz et al. |
| B | 3 | Bel | DQ172320 | 1994 | Nugent et al. |
| B | 3 | Bel | DQ172321 | 1995 | Nugent et al. |
| B | 3 | Bel | DQ172322 | 1997 | Nugent et al. |
| B | 3 | Bel | DQ172323 | 1999 | Nugent et al. |
| B | 3 | Bel | DQ172324 | 1999 | Nugent et al. |
| B | 3 | Bel | DQ172319 | 2003 | Nugent et al. |
| B | 3 | Bel | GU271941 | 2003 | Garre et al. |
| B | 3 | Bel | GU271940 | 2009 | Gryspeerdt et al. |
| B | 3 | Fra | DQ172327 | 2002 | Nugent et al. |
| B | 3 | Hun | HQ654081 | 1997 | Malik et al. |
| B | 3 | Hun | HQ654071 | 2003 | Malik et al. |
| B | 3 | Hun | HQ654068 | 2004 | Malik et al. |
| B | 3 | Hun | HQ654083 | 2006 | Malik et al. |
| B | 3 | Hun | HQ654087 | 2008 | Malik et al. |
| B | 3 | Net | DQ172371 | 1995 | Nugent et al. |
| B | 3 | Net | DQ172372 | 1999 | Nugent et al. |
| B | 3 | Pol | PL_1999_I | 1999 | current paper |
| B | 3 | Pol | PL_2004_I | 2004 | current paper |
| B | 3 | Pol | PL_2008_I | 2008 | current paper |
| B | 3 | UK | DQ172357 | 1983 | Nugent et al. |
| B | 3 | UK | DQ172363 | 1988 | Nugent et al. |
| B | 3 | UK | DQ172367 | 1991 | Nugent et al. |
| B | 3 | UK | DQ172368 | 1993 | Nugent et al. |
| B | 3 | UK | DQ172333 | 2000 | Nugent et al. |
| B | 3 | UK | DQ172334 | 2001 | Nugent et al. |
| B | 3 | UK | DQ172336 | 2002 | Nugent et al. |
| B | 3 | UK | DQ172337 | 2003 | Nugent et al. |
| B | 3 | UK | DQ172339 | 2003 | Nugent et al. |
| B | 3 | UK | DQ172340 | 2003 | Nugent et al. |
| B | 3 | UK | DQ172341 | 2003 | Nugent et al. |
| B | 3 | UK | DQ172342 | 2004 | Nugent et al. |
| B | 3 | UK | DQ172343 | 2004 | Nugent et al. |
| B | 3 | UK | DQ172346 | 2004 | Nugent et al. |
| B | 3 | UK | DQ172348 | 2004 | Nugent et al. |
| B | 3 | UK | DQ172349 | 2004 | Nugent et al. |
| B | 3 | UK | DQ172350 | 2004 | Nugent et al. |
| B | 3 | UK | DQ172351 | 2005 | Nugent et al. |
| B | 3 | Ame | DQ172404 | 1986 | Nugent et al. |
| B | 3 | Ame | DQ172410 | 1990 | Nugent et al. |
| B | 3 | Ame | DQ172411 | 1990 | Nugent et al. |
| B | 3 | Ame | DQ172412 | 1992 | Nugent et al. |
| C | 4 | Bel | GU271939 | 2009 | Gryspeerdt et al. |
| C | 4 | Eth | KP765722 | 2013 | Negussie et al. |
| C | 4 | Eth | KP765723 | 2013 | Negussie et al. |
| C | 4 | Eth | KP765724 | 2013 | Negussie et al. |
| C | 4 | Fra | DQ172326 | 2000 | Nugent et al. |
| C | 4 | Ger | KJ513013 | 2012 | Damiani et al. |
| C | 4 | Hun | HQ654064 | 1990 | Malik et al. |
| C | 4 | Hun | HQ654085 | 2007 | Malik et al. |
| C | 4 | Hun | HQ654086 | 2007 | Malik et al. |
| C | 4 | Ind | KT180205 | 1990 | Anagha et al. |
| C | 4 | Ind | KM285388 | 1998 | Anagha et al. |
| C | 4 | Ind | KM285390 | 2013 | Anagha et al. |
| C | 4 | Ind | KM285391 | 2014 | Anagha et al. |
| C | 4 | Ind | KT180209 | 2014 | Anagha et al. |
| C | 4 | Ind | KT180210 | 2014 | Anagha et al. |
| C | 4 | Ind | KT180211 | 2014 | Anagha et al. |
| C | 4 | Pol | PL_2005_I | 2005 | current paper |
| C | 4 | Pol | PL_2009_II | 2009 | current paper |
| C | 4 | Pol | PL_2013_II | 2013 | current paper |
| C | 4 | Pol | PL_2013_III | 2013 | current paper |
| C | 4 | UK | DQ172354 | 1981 | Nugent et al. |
| C | 4 | UK | DQ172355 | 1981 | Nugent et al. |
| C | 4 | UK | DQ172344 | 2004 | Nugent et al. |
| C | 4 | UK | DQ172345 | 2004 | Nugent et al. |
| C | 4 | UK | DQ172347 | 2004 | Nugent et al. |
| C | 4 | Ame | DQ172407 | 1986 | Nugent et al. |
| D | 5 | Aus | KT324727 | 2003 | Vaz et al. |
| D | 5 | Can | DQ172325 | 1989 | Nugent et al. |
| D | 5 | Ind | KM285386 | 1996 | Anagha et al. |
| D | 5 | Ind | KT180206 | 1996 | Anagha et al. |
| D | 5 | Ind | KT180207 | 1997 | Anagha et al. |
| D | 5 | Ind | KM285387 | 1998 | Anagha et al. |
| D | 5 | Ind | KM285389 | 2007 | Anagha et al. |
| D | 5 | Pol | DQ172373 | 1968 | Nugent et al. |
| D | 5 | Ame | DQ172389 | 1941 | Nugent et al. |
| D | 5 | Ame | DQ172391 | 1972 | Nugent et al. |
| D | 5 | Ame | DQ172392 | 1975 | Nugent et al. |
| D | 5 | Ame | DQ172395 | 1981 | Nugent et al. |
| D | 5 | Ame | DQ172396 | 1981 | Nugent et al. |
| D | 5 | Ame | DQ172398 | 1983 | Nugent et al. |
| D | 5 | Ame | DQ172402 | 1985 | Nugent et al. |
| D | 5 | Ame | DQ172414 | 1999 | Nugent et al. |
| D | 5 | Ame | DQ172415 | 1999 | Nugent et al. |
| D | 5 | Ame | DQ172375 | 2001 | Nugent et al. |
| D | 5 | Ame | DQ172376 | 2001 | Nugent et al. |
| D | 5 | Ame | DQ172377 | 2002 | Nugent et al. |
| D | 5 | Ame | DQ172378 | 2003 | Nugent et al. |
| D | 5 | Ame | DQ172379 | 2003 | Nugent et al. |
| D | 5 | Ame | DQ172387 | 2003 | Nugent et al. |
| E |  | Pol | PL_1999_II | 1999 | current paper |
| E |  | Pol | PL_2002_I | 2002 | current paper |
| E |  | Pol | PL_2003_I | 2003 | current paper |
| E |  | Pol | PL_2004_II | 2004 | current paper |
| E |  | Pol | PL_2004_III | 2004 | current paper |
| E |  | Pol | PL_2006_I | 2006 | current paper |
| E |  | Pol | PL_2007_I | 2007 | current paper |
| E |  | Pol | PL_2009_I | 2009 | current paper |
| E |  | Pol | PL_2010_I | 2010 | current paper |
| E |  | Pol | PL_2010_II | 2010 | current paper |
| E |  | Pol | PL_2011_I | 2011 | current paper |
| E |  | Pol | PL_2012_I | 2012 | current paper |
| E |  | Pol | PL_2012_III | 2012 | current paper |
| E |  | Pol | PL_2012_IV | 2012 | current paper |
| E |  | Pol | PL_2012_V | 2012 | current paper |
| E |  | Pol | PL_2012_VI | 2012 | current paper |
| E |  | Pol | PL_2013_I | 2013 | current paper |
| E |  | Pol | PL_2014_I | 2014 | current paper |
| E |  | Pol | PL_2014_II | 2014 | current paper |
| E |  | Pol | PL_2015_II | 2015 | current paper |
| E |  | Pol | PL_2015_III | 2015 | current paper |
| E |  | UK | DQ172361 | 1986 | Nugent et al. |
| F | 8 | Hun | HQ654075 | 1997 | Malik et al. |
| F | 8 | Hun | HQ654074 | 1998 | Malik et al. |
| F | 8 | Hun | HQ654079 | 1998 | Malik et al. |
| F | 8 | Hun | HQ654067 | 2004 | Malik et al. |
| F | 8 | Hun | HQ654084 | 2006 | Malik et al. |
| G | 7 | Hun | HQ654054 | 1978 | Malik et al. |
| G | 7 | Hun | HQ654055 | 1978 | Malik et al. |
| G | 7 | Hun | HQ654056 | 1978 | Malik et al. |
| G | 7 | Hun | HQ654057 | 1982 | Malik et al. |
| G | 7 | Hun | HQ654059 | 1982 | Malik et al. |
| H |  | Pol | PL_2013_IV | 2013 | current paper |
| H |  | Pol | PL_2013_V | 2013 | current paper |
| H |  | Pol | PL_2013_VI | 2013 | current paper |
| H |  | Pol | PL_2013_VII | 2013 | current paper |
| H |  | Pol | PL_2015_IV | 2015 | current paper |
| I |  | Pol | PL_2001_I | 2001 | current paper |
| I |  | Pol | PL_2006_II | 2006 | current paper |
| I |  | Pol | PL_2010_III | 2010 | current paper |
| I |  | Pol | PL_2012_II | 2012 | current paper |
| J | 6 | UK | DQ172359 | 1985 | Nugent et al. |
| J | 6 | UK | DQ172366 | 1991 | Nugent et al. |
| J | 6 | UK | DQ172335 | 2001 | Nugent et al. |
| K | 3 | UK | DQ172364 | 1989 | Nugent et al. |
| K | 3 | UK | DQ172365 | 1989 | Nugent et al. |
| L | 5 | Ame | DQ172385 | 2003 | Nugent et al. |
| L | 5 | Ame | DQ172388 | 2003 | Nugent et al. |
| M |  | Aus | KF434389 | 1993 | Cuxson et al. |
| M |  | Aus | KT324731 | 1993 | Vaz et al. |
| N | 10 | Hun | HQ654069 | 2004 | Malik et al. |
| O | 9 | Hun | HQ654060 | 1983 | Malik et al. |
| P | 2 | Arg | DQ172309 | 1979 | Nugent et al. |
| Q | 2 | Ame | DQ172408 | 1989 | Nugent et al. |
| R |  | Aus | KF434390 | 1999 | Cuxson et al. |
| S | 2 | Ame | DQ172394 | 1979 | Nugent et al. |
| T |  | UK | DQ172362 | 1987 | Nugent et al. |
| U | 2 | Ame | DQ172384 | 2003 | Nugent et al. |
| V | 4 | UK | DQ172332 | 2000 | Nugent et al. |
| W | 3 | Hun | HQ654073 | 2001 | Malik et al. |
| X |  | Ind | KM285392 | 1990 | Anagha et al. |

^1^ Genetic group as reported in the cited references.

^2^ Polish sequences reported in the current paper are coded with the prefix PL followed by a year of isolation and the number. They have been deposited in GenBank under accession number KY201135-KY201172

^3^ Geographic codes used: Argentina (Arg), Australia (Aus), Belgium (Bel), Canada (Can), Ethiopia (Eth), France (Fra), Germany (Ger), Hungary (Hun), India (Ind), Netherlands (Net), Poland (Pol), United Kingdom (UK), USA (Ame).
